# Life threatening rupture of the diaphragm after Covid 19 pneumonia: a case report

**DOI:** 10.1186/s13019-022-01886-8

**Published:** 2022-06-07

**Authors:** Arvin Imamović, Doris Wagner, Jörg Lindenmann, Nicole Fink-Neuböck, Siegfried Sauseng, Tarik Bajric, Georg Werkgartner, Hans Jörg Mischinger

**Affiliations:** 1 General, Visceral and Transplant Surgery, Medical University of Graz, Auenbruggerplatz 29, 8036 Graz, Austria; 2 Division for Thoracic Surgery, Dept. of Surgery, Medical University of Graz, Graz, Austria

**Keywords:** Covid 19, Cough, Diaphragm, Diaphragmatic rupture, Pneumonia

## Abstract

**Background:**

The incidence of diaphragmatic rupture is low; however, it may be life threatening. Normally caused by blunt trauma, some cases are reported after pulmonary infections with extensive coughing. Covid 19 causes pulmonary infections and pneumonia and has been associated with weakening of the diaphragm after prolonged ventilation. We present a patient who suffered from diaphragmatic rupture 2 months after recovering from a severe Covid 19 pneumonia.

**Case:**

A 71 years old male patient presented with massive thoraco-abdominal pain and severe dyspnea. At the time of admission, the patient was diagnosed with rupture of the diaphragm and developed cardiogenic shock. Intraoperatively there was a 4 cm diameter large rupture of the diaphragm with enterothorax (transverse colon, stomach, spleen, parts of the jejunum). Avulsion of the mesenteric arteries made a segmental resection of the jejunum together with the spleen necessary. A jejuno-jejunostomy was performed and organs were replaced into the abdomen. The rupture of the diaphragm underwent primary closure with non-resorbable suture material. The patient has shown an uneventful post-operative course, fully recovered and was discharged on day 11 after surgery.

**Conclusion:**

Covid 19 is a disease that is known to have various effects on different organs. The diaphragm is only paid heed in case of dysfunction. Also in the setting of Covid 19 it is not known as prominent effector organ. Nevertheless its affection by coughing caused by Covid 19 can lead to life threatening complications.

## Introduction

Car accidents are the most common reason for diaphragmatic rupture which is diagnosed in < 0.5% of trauma cases, 1/3 of which in blunt and 2/3 in penetrating injuries [1, 3]. The left diaphragmatic part is affected in most cases with subsequent enterothorax including the stomach followed by the spleen [2].

There are very rare cases of non-traumatic rupture of the diaphragm requiring surgery after previous operations in the upper abdomen, i.e. liver transplantation, liver resection, gastrectomy and esophageal resection with potential weakening of the diaphragm [4, 5].

The underling pathomechanism for rupture of the diaphragm is a rapid increase of the intraabdominal pressure. Usually, there is a gradient of 7–20 cmH_2_O between the abdomen and thorax. In the case of diaphragmatic rupture, this gradient can exceed 100 cmH_2_O [2]. The most common symptoms include dyspnea, chest pain, abdominal distention, and loss of breath sounds over the affected hemithorax. The latter can be misdiagnosed as pneumonia or pneumothorax [6].

Diaphragmatic dysfunction has been described in patients who had Covid 19 associated pneumonia and needed long time ventilatory support [7]. Ventilator weaning in these patients has been described as often delayed due to ventilatory induced diaphragmatic dysfunction. This can even lead to weaning failure and result in delayed recovery due to lower diaphragmatic excursion and local diaphragmatic thickening with less contractility [8, 9].

Cases on diaphragmatic complications after Covid 19 infections are scarce in the literature. Until now only one case has been reported of diaphragmatic injury without the need for repair [10]. We would like to present a life threatening complication of Covid 19 pneumonia—a patient who suffered a left sided diaphragmatic rupture with herniation of the small intestine and mesenteric avulsion and survived after successful repair. The presented case was written after informed consent of the patient was obtained according to the declaration of Helsinkis guidelines.

## Patient information

A 71 year old male patient was admitted to our tertiary care center from another hospital with dyspnoe and massive thoraco abdominal pain. At arrival he presented with cardio respiratory instability (125 bpm average heart rate with blood pressure of 95/45 mmHg) requiring positive inotrope support to maintain at least these values using high dose noradrenaline (Arterenol 0,14 yg/kg bodyweight). No family history on pulmonary diseases was present nor any active Covid 19 infections in the patients close environment had been reported.

His main concern was that he presented with massive abdominal and thoracic pain that had started on transport to our center which took approximately 90 min.

### Timeline

**Table:** 

February 25th, 2021, 19:00	Transferred to our hospital via air ambulance in acute respiratory distress with acute onset
Feburary 25th, 2021, 19:20	Acute CT scan thorax and abdomen revealing the herniation
February 25th, 2021, 20:30	Start of operative procedure and postoperative intensive care stay
February 28th 2021	Extubation and transfer to normal ward
March 12th 2021	Transfer to admitting hospital

### Diagnostic assessment

**Fig. 1:**
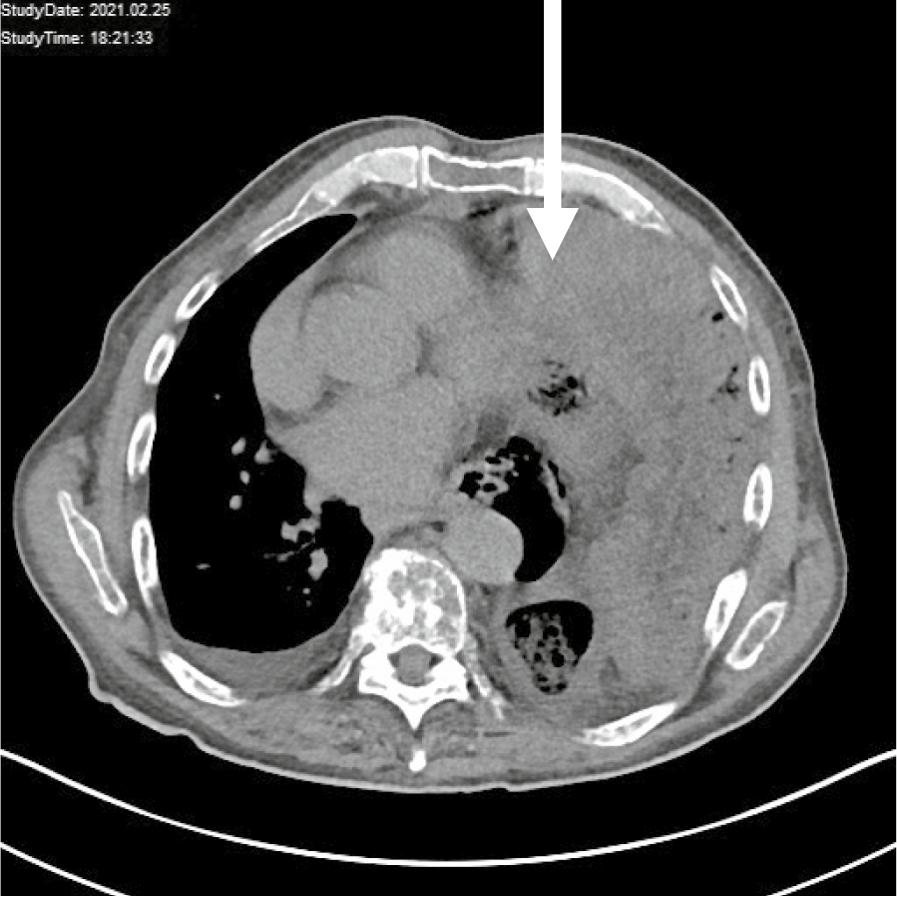
preoperative CT imaging revealed a massive diaphragmatic herniation into the left thorax. As visible on the CT image dextrocardia was present and abdominal organs compressed the left lung resulting in massive dyspnoea of the presented patient (arrow)

Apart from elevated white blood cells (16.3 G/L Ref: 4.4–11.3 G/L) and elevated lactate (2.9 mmol/L Ref: 0.5–2.2 mmol/L) the patient suffered from dyspnea which was reflected in respiratory acidosis (pH 7.107, pO2 16.6 mmHg, pCO2 68.9, HCO3 21.7 mmol/L, Base Excess − 8.3 mmol/L). After initial CT scan the patient was transferred to the operation room immediately. The thoraco abdominal computed tomography scan revealed a left sided diaphragmatic rupture with herniation of the left hemicolon as well as of parts of the jejunum. The diaphragmatic gap was measured with a diameter of 4 cm on preoperative CT scan. (Fig. 1).

### Patient history

The patient had undergone Covid 19 infection 4 months prior to his admission showing the diaphragmatic rupture. During his Covid 19 infection the patient had been continuous positive airway pressure support for 5 days but had never required invasive ventilatory support leading to intubation. The Covid 19 infection lasted for 19 days in this patient, requiring 8 days of ICU care and subsequent 11 days of regular hospital treatment. The patient had fully recovered from his Covid 19 infection but reported continuous coughs as residual effects after severe Covid during the period between the infection and the admission to our clinic.

### Therapeutic intervention

Operative exploration via laparotomy showed an approximately 7 cm wide diaphragmatic rupture at the left lateral end of the diaphragm with herniation of the stomach, the spleen, the transverse colon as well as parts of the jejunum. Incarceration was present for the jejunal parts and the mesenteric arteries of the affected jejunal segments were avulsed resulting in massive haemorrhage into the abdomen. After bleeding control was achieved with clamping and stitches, the diaphragmatic hole was enlarged in the direction of the esophageal hiatus, the displaced organs were removed and relocated in the abdominal cavity. Two jejunal segments had to be resected with jejuno-jejunal reconstruction using PDS 4/0 continuous sutures for the two layered anastomosis. The stomach as well as the transverse colon recovered and did not require surgical treatment. The spleen was resected after re location into the abdomen. Diaphragmatic reconstruction was performed with direct repair using Ethibond 1/0 sutures and a thoracic drain was placed on the left side. After decompression of the thorax and closure of the diaphragm the patients cardio respiratory situation stabilized.

### Follow up and outcomes

Postoperatively the patient was transferred to an ICU and remained on inotropic support for 24 h and required ventilatory support for 36 h postoperatively. He was extubated successfully and was eventually transferred into the primary care hospital on postoperative day 10. He remained stable after the operation and did not require any more interventions. He was discharged from the primary referral hospital after another 10 days of recovery. The patient is well and alive after the diaphragmatic repair.

## Discussion

COVID 19 can mimic several clinical scenarios ranging from flulike symptoms dry cough and sore throat to severe acute respiratory distress syndrome (ARDS) and multi-organ failure with death. Cough has already been associated with diaphragmatic injuries in old and frail patients [11]. Long term ventilatory support is often associated with weakening of the diaphragm especially if the weaning phase is longer—like in Covid associated pneumonia requiring intubation and ventilation [12]. Sometimes the defects at the diaphragm are caused by excessive coughing [13]. The presented patient had had a Covid associated pneumonia 4 months prior presentation and had recovered fully before his referral due to the upper mentioned symptoms. As a consequence of the pneumonia itself, of the prolonged ventilation and of the extensive coughing the patient may complain of chest and abdominal pain and even might have had an asymptomatic diaphragmatic hernia. Abdominal organ herniation into the chest can impair ventilation and oxygen delivery. This was an extremely serious situation in our patient and was extremely complex to diagnose and manage. Chest X-rays prior to the presentation with the diaphragmatic herniation did not reveal any signs of diaphragmatic rupture in this patient.

Despite the fact that at first sight this patient might seem like an ordinary patient, needing surgical treatment, the fact that this life threatening diaphragmatic rupture is in direct relation to the Covid infection is novel to the literature. Besides the over 6 million fatalities Covid 19 caused during the past two years, long term effects after infection become more and more focus. The diaphragmatic weakness resulting in rupture might be one of these long term effects – and so, even if it is a rare one – of relevance.

To the best of our knowledge only one case of Covid 19 associated diaphragmatic rupture exists until now in the literature. Our patient did not have an active Covid 19 associated pneumonia when he presented with the thoraco abdominal pain but rather presented with the diaphragmatic rupture as late consequence of the underwent infection. The incidence of abdominal organ herniation due to diaphragmatic rupture is unknown since many cases likely go undiagnosed. Diaphragmatic rupture is a life-threatening condition which need prompt diagnosis and repair. CT scanning is the gold standard technique to evaluate the diaphragm and this is often not done in Covid 19 cases. Chest X-ray might be too imprecise to diagnose this condition. Our special emphasis in this case lies on creating awareness for this condition as well as presenting a special life threatening complication yet rare and not widely reported complication of Covid 19.

### Patient perspective

The patient stated multiple times while on the ICU and on the normal ward how grateful he was that he was able to breath again. He was discharged in good health from his primary referral hospital and stated in a phone call with Wagner D in November 2021 that he was still in good health and had recovered his full shape in summer 2021.

## Data Availability

Data is available upon request via the email address of the corresponding author: doris.wagner@medunigraz.at.

## Abbreviations

CT: Computed tomography
ICU: Intensive care unit
